# Idiopathic intramural hematoma of the right colon. A case report and review of the literature

**DOI:** 10.1016/j.ijscr.2019.05.004

**Published:** 2019-05-09

**Authors:** Rosario Vecchio, Emma Cacciola, Michele Figuera, Renato Catalano, Giuseppe Giulla, Emanuele Rosario Distefano, Eva Intagliata

**Affiliations:** a Department of General Surgery and Medical-Surgical Specialties, Policlinico – Vittorio Emanuele Hospital, University of Catania, Italy; b Department of Medical, Surgical Sciences and Advanced Technologies, Policlinico – Vittorio Emanuele Hospital, University of Catania, Italy; c Department of Radiology, Policlinico – Vittorio Emanuele Hospital, University of Catania, Via S. Sofia, 78 – 95100 Catania, Italy

**Keywords:** Intestinal hematoma, Large bowel hematoma, Intestinal obstruction, Mesenteric hemorrhagic effusion, Case report

## Abstract

**Introduction:**

Intestinal hematoma is usually observed after an abdominal trauma or in patients treated with anticoagulant therapy. Conversely, idiopathic bowel hematoma is very rare, being colon involvement sporadic with few reports in the Literature.

**Presentation of case:**

The Authors report a case of idiopathic spontaneous large bowel hematoma. A 48-year-old man was admitted for a thoraco-abdominal pain and signs of acute abdomen and fever. After ruling out heart diseases, a CT scan revealed a marked thickening of the ascending colon wall, obstructing the bowel lumen. Leukocytosis was observed. In an emergency setting, an explorative laparotomy was performed. Hemoperitoneum and a large hematoma involving the caecum and the ascending colon were detected, together with intramesenteric and retroperitoneal blood effusion. A right hemicolectomy was accomplished. Histopathology confirmed the diagnosis of large bowel hematoma. Post-operative molecular diagnostic testing for coagulative disorders failed to demonstrate any genetic variation associate with hemorrhagic predisposition.

In the post-operative course, the patient experienced a left basal bronco-pneumonia with increased unilateral pleural effusion, successfully treated by a thoracic drain and antibiotic therapy.

**Discussion:**

The reported case and Literature data show that diagnosis of idiopathic colon intramural hematoma is challenging, especially in the emergency setting. Although conservative therapy is the first line treatment, surgery still has an important role when the diagnosis is uncertain, medical treatment fails or a complication, such untreatable bleeding, perforation or occlusion occur.

**Conclusion:**

The Authors report a very rare case of spontaneous intramural hematoma of the right colon. Surgery still has a role in selected cases.

## Highlights

• A very rare case of spontaneous colon hematoma has been reported.
• The rarity of our case of colon hematoma is due to the fact that it is idiopathic.
• The topic is still under discussion in the Literature since the pathophysiology remains still unknown.

## 1 Introduction

Intramural hematoma of the bowel has been most commonly recognized as a complication of blunt trauma [1] or as a consequence of anticoagulant therapy [2].

Other risk factors are hemophilia [3], leukemia, lymphoma, chemotherapy, idiopathic thrombocytopenic purpura [4]. Spontaneous idiopathic occurrence is, on the contrary, very rare and involvement of the colon is exceptional with only sporadic cases reported in the Literature [5,6].

Because of the rarity of spontaneous idiopathic large bowel hematoma (SILBH), often the diagnosis is not suspected. Radiologic imaging may be misleading [6]. Furthermore, there is little information about the appropriate treatment, which has been either non-operative or surgical in the rare cases reported in the Literature [[6], [7], [8]]. We report a unique case of right colon intramural hematoma of unknown etiology, with an atypical clinical presentation. A review of the Literature has been made, and diagnostic and therapeutic management options are discussed.

The work has been reported in line with the SCARE criteria [9].

## 2 Case report

**Fig. 1:**
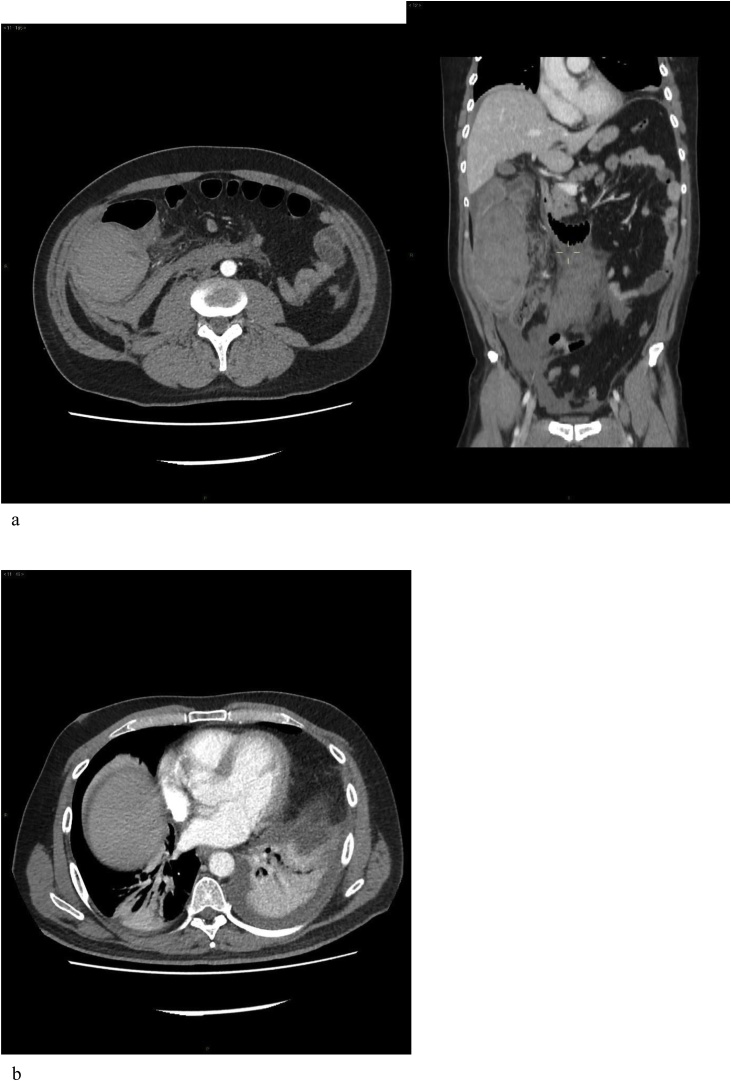
(a) CT scan revealing marked thickening of right colon. (b) Pleural effusion with pulmonary atelectasia.

A 48–year–old–man was admitted to the hospital in an emergency setting because of thoracic and abdominal pain. No other significant relevant past medical or mental illness was reported. At the admission, an X-ray showed a right pleural effusion. A ST elevation myocardial infarction (STEMI) was suspected at ECG, but a myocardial ischemia was ruled out on a coronary angiography. The day after the hospitalization, the patient developed signs and symptoms of acute abdomen, intestinal occlusion and fever. Because of a palpable mass in the right quadrants of the abdomen, a CT scan was performed (Fig. 1a). A marked thickening of the ascending colon wall, from the caecum up to the right colonic flexure, with abundant intraluminal blood density-material was demonstrated. Concomitant marked thickening of the anterior para-renal fascia with presence of retroperitoneal effusion and peritoneal effusion were also detected (Fig. 1b). A moderate pleural bilateral effusion was confirmed with associated pulmonary basal atelectasis. With laboratory findings showing elevated white blood cells count (15.08 × 10^3^ /μL) and PCR >500 mg/L, an intra-abdominal infection was suspected and the patient was at this time referred to surgery for urgent abdominal exploration.

**Fig. 2:**
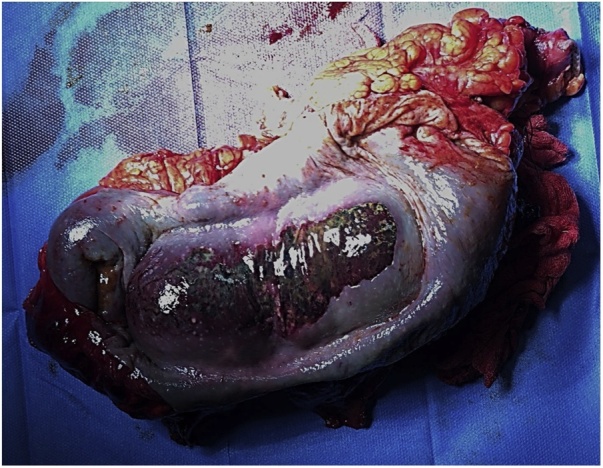
Surgical specimen showing massive intramural hematoma of the right colon.

A midline laparotomy was accomplished. At initial exploration, hemoperitoneum was observed. Intramesenteric and retroperitoneal hemorrhagic effusion was also evident. The right-colon was involved by a massive intramural hematoma (Fig. 2), which dissociated all the bowel layers from the serosa to the mucosa with initial evidence of perforation. A right hemicolectomy was performed in a traditional fashion with a side-to-side ileo-transverse colon anastomosis.

Histopathology findings of the surgical specimen showed a transmural hematoma without any other underlying macro- or microscopic alterations. Post-operative molecular diagnostic testing for coagulative disorders failed to demonstrate any genetic variation associated with hemorrhagic predisposition, revealing only genetic variations and polymorphism associated with predisposition to thrombophilia.

In the postoperative course, the patient experienced a left basal bronco-pneumonia with increased unilateral pleural effusion that required a thoracic drain. Treated with antibiotic therapy, the patient was discharged and healed on postoperative day 12.

## 3 Discussion

The first report of an intestinal hematoma was published in 1838 [10] by McLauchlan, who described a traumatic duodenal obstruction hematoma at autopsy. Later, Sutherland in 1904 [11] and von Khautz in 1908 [12] reported non-traumatic cases of intestinal hematoma in patients respectively affected by a Henock-Schonlein purpura and hemophilia. After these initial observations, intestinal hematoma has been reported in case of trauma, in patients with bleeding disorders, malignancies, anticoagulant therapy or chemotherapy, vasculitis, or after bone marrow transplantation [13,14].

Idiopathic cases, with unknown or uncertain origin, have also been described but their occurrence has been reported in less than 5% of cases [2,8,[15], [16], [17], [18], [19], [20]]. Intramural hematoma may occur from esophagus to rectum [17], being the duodenum the most common site [19,21]. Intramural isolated hematoma of the colon is a very rare disease [16,22], much less frequent than hematoma in other segments of gastrointestinal tract [[23], [24], [25]]. It can be supposed that the less commonly involvement of the colon may be due to the protective role of the Teniae coli, which could prevent the diffusion of the hemorrhage in the bowel wall [4]. The occurrence of large bowel hematoma of idiopathic origin is very rare, with only sporadic cases reported in the Literature [5,6]. In our report, no risk factors for bleeding have been identified and in the absence of an etiology, the case has been classified as sporadic, very rare, idiopathic case.

Most patients with intestinal hematoma complaint of abdominal discomfort or pain for several days before developing symptoms of intestinal obstruction, which is the most frequent clinical picture of intestinal hematoma at the time of diagnosis [15]. In Abbas’ series, obstruction has been observed in 11 out of 13 patients [4]. Intestinal perforation, bleeding and hemoperitoneum might be other clinical manifestations [26]. Progression of symptoms and subsequent development of intestinal obstruction may be related to the continuous bleeding itself or it may be secondary to an osmotic effect that draws fluids from the surrounding structures. When hematoma, usually starting from the submucosa, involves all the intestinal layers, hemoperitoneum can be observed. This finding has been commonly reported in cases of small bowel hematoma and it seems to be secondary to leakage of blood from a thickened and inflamed bowel wall. In intramural isolated hematoma of the colon, symptoms are less pronounced, probably because of the large lumen that makes more difficult to have an intestinal obstruction. In our reported case, hematoma was so extended that an intestinal obstruction developed. Moreover, we observed intraoperatively an associated intramesenteric and retroperitoneal hemorrhage that is uncommon in the bowel hematoma described in the Literature [4].

Laboratory tests may reveal anemia and leukocytosis. Abba et al. observed leukocytosis in 13 out of 13 patients observed with intestinal hematoma [4]. In Hughes et al. review [27], elevation of white blood cells were reported in 72% of the patients. Unlike anemia, leukocytosis is difficult to explain. It has been emphasized that it is probably due to the hemorrhagic disruption of the intestinal wall, with intramural and/or peritoneal diffusion of intestinal bacteria with subsequent infections. This hypothesis, however, is not confirmed in our case by the pathologic findings, that failed to demonstrate any sign of inflammation at the surgical specimen. It has to be remarked that leukocytosis might be related to an associated medical disease with sepsis. Moreover, leukocytosis with a mass observed at CT scan might have difficult the diagnosis of hematoma, since an intra-abdominal infection is difficult to rule out in this case.

Diagnosis of intestinal hematoma may be challenging. In the emergency setting, when symptoms of obstruction or perforation are suspected, a plain abdominal X-ray is helpful. It may reveal typical patterns of bowel obstruction or signs of intestinal perforation. Colonoscopy performed by an experienced endoscopist in not complicated cases may be useful but not diriment, showing blue round and/or erythematous formations in the submucosal layer with a submucosal mass obstructing the luminal space [15,16,28]. Biopsy can be helpful in case of suspicious of intraluminal tumor, but the tissue contained in hematoma is very friable and bloody [16]. Contrast-enhanced CT scan allows detailed diagnosis [15,22,28,29] and it is essential for the definitive diagnosis. In Abbas’ report [4], CT scan was diagnostic in all patients. Imaging characteristics include wall thickening. Besides bowel thickening, in our case signs at CT scan of fluid diffusion in retroperitoneum or intraperitoneally were observed. Although CT scan is diagnostic in the majority of cases, differential diagnosis with other benign or malignant bowel diseases particularly in obstructed or perforated patients might be however difficult.

In past reports of intestinal hematoma, surgery has been commonly performed for abdominal diagnostic exploration with resection or bypass of the intestinal involved segment. Nowadays, for diagnosis of bowel hematoma explorative laparotomy is seldom needed, since radiologic imaging studies usually are accurate [4]. When abdominal trauma or anticoagulant therapy are not present in the clinical history of the patient, radiologic imaging might be misleading in the diagnosis, especially when large bowel is the site of hematoma [6,18].

Nozu et al. reported a very rare case of idiopathic intramural hematoma of the colon in an 82-year-old man who did not have history of trauma or anticoagulant intake. He presented abdominal discomfort and constipation, slight anemia and laboratory findings of inflammation. In this case, enhanced CT scan was not diriment and colonoscopy could not detect the lesion. Laparotomy was performed since a neoplastic disease could not be ruled out [6].

It has to be noted that diagnosis is even more difficult to achieve in the emergency setting. In this situation, explorative laparotomy remains strongly indicated not only for diagnosis, but also for treatment. In the patient we report, preoperative diagnostic work-up was not diriment and an intra-abdominal infection with reactive pleural effusion was suspected. Surgery was performed anyway to treat the obstruction of uncertain etiology.

It should be emphasized that today if a definitive diagnosis of colon intramural hematoma is performed in patients with anticoagulant therapy, and symptoms and clinic signs showing urgent surgery are absent, surgery is not routinely indicated and conservative therapy must be accomplished [26]. Discontinuation of anticoagulant therapy might solve intestinal hematomas in 30% of cases [15].

Fernandes et al. reported a case of spontaneous intramural hematoma treated conservatively. Suspension of anticoagulant therapy and complete healing within one month of a descending colon hematoma was observed in a 73-year-old male patient who was taking warfarin and aspirin for cardiac pathology [28].

Liu et al. admitted a 57-year-old male patient with an intramural hematoma of the sigmoid colon, diagnosed at enhanced CT scan. A conservative treatment by means of total parenteral nutrition, blood transfusion and nose-gastric suction was performed, and the patient healed within 3 weeks [22].

Kwon et al. reported a case where a 62-year-old woman in treatment with warfarin and ticlopidine because of artificial cardiac valves, presented with bloody stools and lower abdominal cramping. The physical examination revealed mild tenderness in the left lower abdominal quadrant without peritoneal irritation. She was successfully treated conservatively by means of heparin and fasting [16]. Lobo et al. achieved similar results after conservative treatment in a 63-year-old patient affected by colon hematoma [18].

Surgery still may have a role if conservative therapy fails and the lesions do not spontaneously heal within a few week. It can be performed either by open or laparoscopic approach [30]. Some Authors have observed that relief of the dabbing effect during conservative treatment could lead to further bleeding and could increase the risk of infective complications [17,21].

Thomas et al. described a case of colonic obstruction caused by intramural hematoma in a 74-year-old woman with intake of warfarin. Warfarin was stopped, but because of worsening abdominal signs and symptoms, right hemicolectomy was performed. The patient died subsequently because of pneumonia [29].

Sakamoto et al. reported a 35-year-old man with a CT scan diagnosis of a large hematoma in the ascending colon. After an initial conservative treatment, a new CT scan performed for worsening of clinical conditions showed an intestinal perforation. An urgent laparotomy with right hemicolectomy was performed. Histologic examination showed an intramural bleeding that caused wall ischemia and subsequent perforation [19].

Yu et al. reported a case where a 70-year-old woman with abdominal tenderness at the left lower quadrant 3 days after coronaric stent implantation, a CT scan allowed the diagnosis of submucosal hematoma. After two days of conservative treatment, abdominal pain and distention along with signs of peritoneal irritation get worse. Urgent sigmoidectomy was performed. Pathological examination showed colonic hemorrhage with necrosis [20].

Zhou et al. reported a case of a 70-year-old woman who developed a bleeding submucosal hematoma at the sigmoid colon. Conservative treatment failed to stop the bleeding and a left hemicolectomy was finally performed [2].

In conclusion, our reported case and Literature data show that diagnosis of colic intramural hematoma is challenging when a clinical history with trauma or anticoagulant therapy cannot be demonstrated. Furthermore, diagnosis of intestinal hematoma is difficult to achieve in an emergency setting, since in this condition it is challenging to rule out other obstructing or perforated colon diseases. Conservative treatment is the first choice. However, surgery still has an important role when the diagnosis is uncertain, medical treatment fails or a complication, such as intractable bleeding, perforation or occlusion occur.

## Conflicts of interest

There is no conflict of interest.

## Sources of funding

There are no sources of funding.

## Ethical Approval

The study is exempt from ethical approval in our Hospital in Italy.

## Consent

Written informed consent was obtained from the patient for publication of this case report and accompanying images. A copy of the written consent is available for review by the Editor-in-Chief of this journal on request.

## Author contribution

All the Authors (Vecchio R, Cacciola E, Figuera M, Catalano R, Giulla G, Distefano R, Intagliata E) contributed to conceptualization, data curation, investigation, methodology and writing.

Vecchio R and Intagliata E, in addition, supervised and reviewed the manuscript.

## Registration of Research Studies

None.

## Guarantor

Dr. Eva Intagliata.

## Provenance and peer review

Not commissioned, externally peer-reviewed.
